# Effectiveness of High-Flow Nasal Oxygen vs. Standard Nasal Oxygen in Preventing Hypoxia During Procedural Sedation in Minor and Moderate Burn Patients: A Prospective Observational Study

**DOI:** 10.3390/medicina62081432

**Published:** 2026-07-23

**Authors:** Sümeyye Demirhan, Cihan Döğer, Bilge Tuncer, Ezgi Erkılıç

**Affiliations:** Department of Anesthesiology and Reanimation, Ankara Bilkent City Hospital, Ankara 06800, Türkiye; dmrhnsmyy@gmail.com (S.D.); cihandoger@gmail.com (C.D.); eerkilic72@yahoo.com (E.E.)

**Keywords:** high-flow nasal oxygen, standard nasal oxygen, procedural sedation, burn patients, hypoxia, BIS monitoring, NIRS

## Abstract

*Background and Objectives*: Hypoxia is the most common adverse event in patients undergoing procedural sedation. Burn-related pain is severe and often requires high doses of opioids and anxiolytics, increasing the risk of respiratory depression. This study aimed to compare the effectiveness of high-flow nasal oxygen (HFNO) and standard nasal oxygen (SNO) in preventing hypoxia during deep sedation in patients with minor and moderate burns. *Materials and Methods*: This single-center prospective observational study enrolled 76 adult patients (ASA I–III) with minor to moderate burns undergoing supine-position procedures under sedation. Patients were allocated to HFNO (n = 38) or SNO (n = 38) groups according to a pre-specified, non-randomized allocation rule based on an alternating/sequential assignment by admission order. Peripheral oxygen saturation (SpO_2_), Bispectral Index (BIS), and Near-Infrared Spectroscopy (NIRS) were monitored throughout. Desaturation was defined as SpO_2_ ≤ 90%; severe hypoxia as SpO_2_ < 75%. Sedation depth was standardized using BIS (target: 60–80). Propofol–ketamine-based induction was used in all patients. *Results*: Groups were comparable in demographics, comorbidities, burn characteristics, and procedural duration. SpO_2_ ≤ 90% occurred in 79% of SNO patients vs. 11% in the HFNO group (*p* < 0.001). SpO_2_ between 75 and 90% was observed in 68% of SNO vs. 0% of HFNO patients (*p* < 0.001), and SpO_2_ < 75% in 26% vs. 0% (*p* < 0.001). Airway interventions—jaw thrust, flow increase, and FiO_2_ increase—were significantly more frequent in the SNO group (84% vs. 2.6%, 84% vs. 11%, and 84% vs. 5.3%, respectively; all *p* < 0.001). No significant between-group differences were noted in procedure interruptions, laryngospasm, BIS, NIRS, or hemodynamic parameters. *Conclusions*: HFNO significantly reduced the incidence of hypoxia and the need for airway interventions compared with SNO during deep sedation in minor and moderate burn patients. These findings suggest that HFNO may represent a preferable oxygenation strategy in this population; given the observational design of this study, confirmation in randomized controlled trials is warranted before this can inform routine practice recommendations.

## 1. Introduction

Pain caused by burns is severe and difficult to manage. In the anesthetic management of burn patients, high doses of opioids and anxiolytics are often required to achieve deep sedation [1]. However, anesthetic agents used during sedation—such as benzodiazepines, propofol, and opioids—can cause respiratory depression, making patients more susceptible to hypoventilation and hypoxemia due to airway obstruction [2].

Standard nasal oxygen (SNO) is one of the most commonly used methods of oxygen delivery. It is preferred because it is lightweight and does not interfere with basic patient functions such as speaking, coughing, and eating. With this method, oxygen can be delivered at flow rates of 1–6 L/min, yielding a fraction of inspired oxygen (FiO_2_) ranging from 24% to 45%. However, it offers no active humidification and may cause mucosal dryness and reduced patient tolerance at flow rates above 4 L/min [3].

High-flow nasal oxygen (HFNO) provides an adjustable high gas flow of 40–60 L/min, delivering heated and humidified oxygen at concentrations ranging from 21% to 100%. By generating a positive pressure effect, it helps maintain alveolar patency, reduces the work of breathing, clears airway dead space, and prevents CO_2_ accumulation [4,5]. Although HFNO is frequently used in intensive care units, its adoption in operating rooms and non-operating-room sedation settings remains limited [6].

Studies on HFNO use during procedural sedation have predominantly been conducted in gastrointestinal endoscopy units. Evidence in burn patients—who require higher analgesic doses and are thus at greater risk of respiratory depression—is scarce and largely limited to case series [4,7].

Burn patients differ substantially, in several physiological respects, from the endoscopy and bronchoscopy populations in which HFNO has been most extensively studied. Extensive fluid shifts, a hypermetabolic and hyperinflammatory state, altered drug pharmacokinetics, and the considerably higher analgesic and sedative requirements driven by severe procedural pain place burn patients at elevated risk of sedation-related respiratory depression. These physiological differences limit direct extrapolation of endoscopy-derived evidence to burn care and reinforce the need for burn-specific data such as that provided by the present study.

The aim of this study was to compare the effects of HFNO and SNO on hypoxia prevention in minor and moderate burn patients undergoing deep sedation, using BIS and NIRS monitoring.

## 2. Materials and Methods

This prospective observational study was conducted between July 2024 and January 2025 following ethical approval (No. 6462, date 7 February 2024) from the Ministry of Health of the Republic of Türkiye, Ankara Bilkent City Hospital Ethics Committee. The ClinicalTrials.gov registration number is NCT06756906.

### 2.1. Study Population

A total of 76 adult volunteer patients (≥18 years) with ASA physical status I–III, of either sex, scheduled for supine-position procedures under sedation in the Burn Unit of Ankara Bilkent City Hospital with minor-to-moderate burns, were enrolled. Patients were assigned to the HFNO or SNO group according to a pre-specified, non-randomized allocation rule based on an alternating/sequential assignment by admission order. The treating clinician did not select the oxygenation method based on the anticipated respiratory risk of the individual patient. Written informed consent was obtained from all participants. One patient in the HFNO group was excluded due to intolerance to sedation requiring conversion to general anesthesia, resulting in 38 patients per group.

Exclusion criteria were: age < 18 years, ASA ≥ IV, pregnancy, chronic respiratory disease, tracheostomy, major burns, inhalation injury, or facial burns.

### 2.2. Monitoring

Upon admission to the procedure room, all patients underwent continuous monitoring of systolic arterial pressure (SAP), diastolic arterial pressure (DAP), mean arterial pressure (MAP), electrocardiography (ECG), SpO_2_, BIS (Covidien, Medtronic BIS™ 185-051 Covedien IIc, Mansfield, MA, USA), and NIRS (INVOS™ 5100C Cerebral/Somatic Oximeter, Medtronic; Minneapolis, MN, USA). Hemodynamic and monitoring parameters were recorded before the procedure and at 5 min intervals throughout.

### 2.3. Oxygenation Protocols

SNO group: Patients were preoxygenated for 3 min at 8 L/min before induction; 5 L/min was maintained during the procedure.

HFNO group: Using Inspired O_2_FLO™, preoxygenation was performed at 100% FiO_2_, 40 L/min, 36 °C for 3 min. During the procedure, flow was set to 50 L/min with 50% FiO_2_.

### 2.4. Sedation Protocol

One minute before induction, all patients received 1 mg intravenous (IV) midazolam and 0.5–1 µg/kg fentanyl as premedication, followed by 10 mg IV lidocaine. Induction was achieved with 0.5–1 mg/kg 1% propofol and 0.5–1 mg/kg ketamine. Propofol infusion (2–4 mg/kg/h) was titrated to maintain BIS values between 60 and 80; propofol was discontinued when BIS < 60. Total drug doses and procedure duration were recorded.

Hypotension (≥25–30% decrease from baseline blood pressure) was treated with a fluid bolus or IV ephedrine (5–10 mg). Bradycardia (HR < 50 bpm) was treated with 0.5 mg IV atropine.

### 2.5. Outcome Measures

The following events were recorded: SpO_2_ categories (=90%, 75–90%, ≤75%), airway interventions (jaw thrust, flow rate increase, FiO_2_ increase), procedure interruptions, and laryngospasm. SpO_2_ ≤ 90% was defined as desaturation; SpO_2_ < 75% was defined as severe hypoxia (requiring bag–mask ventilation). We selected 75% rather than the 80–85% thresholds more commonly used in the procedural sedation literature because, in our protocol, this level corresponded to the point at which bag-mask ventilation was clinically required; we therefore consider it a clinically actionable rather than an arbitrary statistical cut-off. This stricter definition limits direct numerical comparison of severe-hypoxia incidence with studies that use higher thresholds, a point also noted by Turnbull in relation to the wide variability of hypoxia definitions across the procedural sedation literature [8]. In case of desaturation (SpO_2_ ≤ 90%), the patient was verbally stimulated by the attending anesthesiologist to breathe. When low saturation persisted, drug doses were reduced by applying chin lift and chin thrust maneuvers, respectively. Balloon-mask ventilation was performed by the attending anesthesiologist when SpO_2_ decreased to <75%. Lowest SpO_2_, BIS, and NIRS values were noted. The Modified Aldrete Score was assessed at 0 and 15 min post-procedure; patients with a score > 8 were transferred to the ward.

### 2.6. Statistical Analysis

Quantitative variables are presented as mean ± SD or median (Q1, Q3) as appropriate. Normality was assessed by histogram inspection. Independent-group comparisons used Student’s *t*-test, Mann–Whitney U test, Pearson’s χ^2^, Fisher’s exact test, or Yates-corrected χ^2^ test as appropriate. Repeated measurements were analyzed with Repeated Measures ANOVA or the Wilcoxon signed-rank test.

Mixed linear models with random intercepts were used to evaluate the effect of oxygenation type on BIS and NIRS, adjusting for age, sex, ASA score, BMI, and procedure duration. Multiple logistic regression was used to assess the association between propofol dose and oxygenation method, adjusting for BMI, ASA score, procedure duration, and smoking status. Linear regression and polynomial spline (p-spline) models were applied to evaluate the relationship between SpO_2_ and percentage changes in BIS and NIRS. Statistical significance was set at *p* < 0.05. All analyses were conducted using RStudio Version 2024.12.0+467.

### 2.7. Power Analysis

Sample size was calculated using G*Power 3.1.9.7. Assuming a desaturation incidence of 48% in the SNO group and 15% in the HFNO group (α = 0.05, power = 80%), a minimum of 70 patients (n_1_ = 35, n_2_ = 35) was required. A target of 76 patients (38 per group) was enrolled to account for potential dropouts.

## 3. Results

### 3.1. Demographic and Baseline Characteristics

No statistically significant differences were observed between the groups regarding sex, age, height, weight, BMI, or ASA score (all *p* ≥ 0.3) (Table 1). Similarly, comorbidities and smoking status were comparable between groups (all *p* ≥ 0.2) (Table 2).

### 3.2. Burn Characteristics and Procedural Data

No significant differences were identified between groups in burn type, burn percentage, or procedure type (all *p* ≥ 0.6) (Table 3). Scald burns were most common, followed by flame burns. Total propofol dose and procedure duration were comparable between groups (Table 4).

### 3.3. Oxygen Saturation

**Figure 1:**
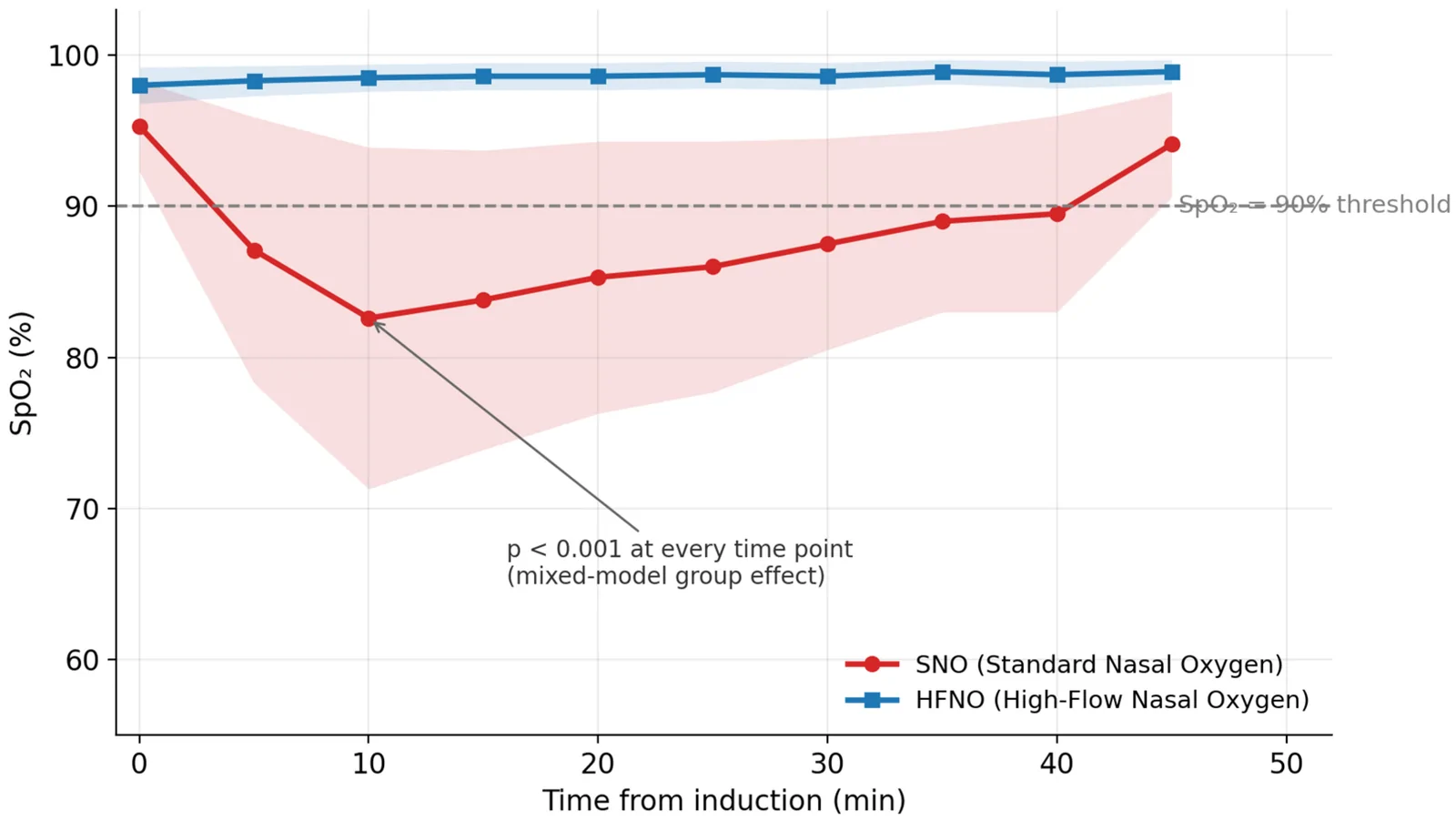
SpO_2_ values over time in the HFNO (blue) and SNO (red) groups. Data are expressed as mean ± SD. The dashed horizontal line indicates the 90% desaturation threshold. HFNO: high-flow nasal oxygen; SNO: standard nasal oxygen; SpO_2_: peripheral oxygen saturation.

SpO_2_ decreases were significantly less frequent in the HFNO group across all severity categories (all *p* < 0.001) (Table 5). SpO_2_ values in the HFNO group remained above 90% and were stable throughout the procedure, whereas marked desaturations (SpO_2_ < 75%) were observed in the SNO group, particularly in the early minutes of the procedure (Figure 1).

### 3.4. Airway Interventions

Jaw thrust, flow rate increase, and FiO_2_ increase were performed significantly more frequently in the SNO group (all *p* < 0.001). Jaw thrust was required in 84% of SNO patients vs. 2.6% in the HFNO group; flow rate increases in 84% vs. 11%; FiO_2_ increases in 84% vs. 5.3%. No significant between-group differences were found for procedure interruptions or laryngospasm (Table 6).

### 3.5. BIS and NIRS

**Figure 2:**
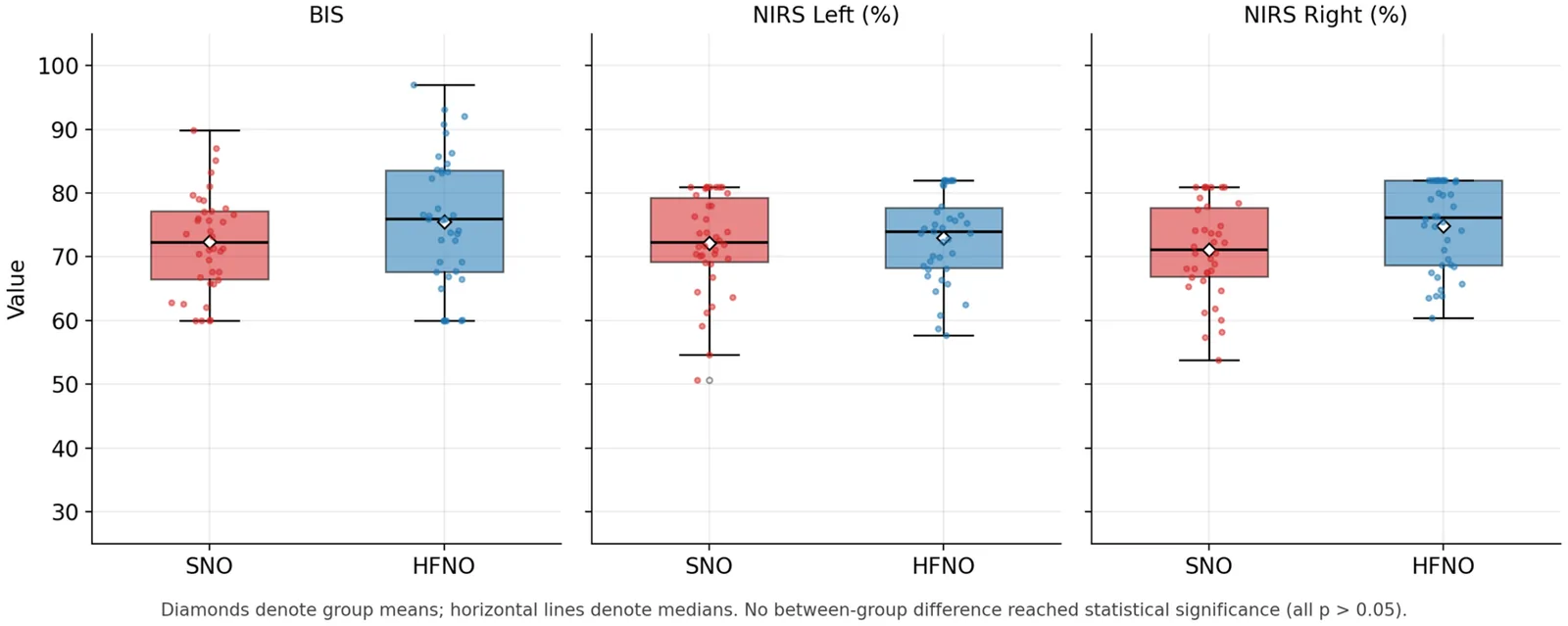
Distribution of BIS, NIRS Left, and NIRS Right values by oxygenation group. Boxes represent the interquartile range; horizontal lines indicate the median; whiskers extend to the most extreme non-outlier values; diamonds denote group means. BIS: bispectral index; NIRS: near-infrared spectroscopy; HFNO: high-flow nasal oxygen; SNO: standard nasal oxygen.

Although BIS values tended to be slightly lower in the HFNO group, this difference was not statistically significant, indicating comparable depths of sedation. NIRS values were higher in the HFNO group but also did not reach statistical significance. The distribution of BIS, NIRS Left, and NIRS Right by oxygenation group is shown in Figure 2.

**Figure 3:**
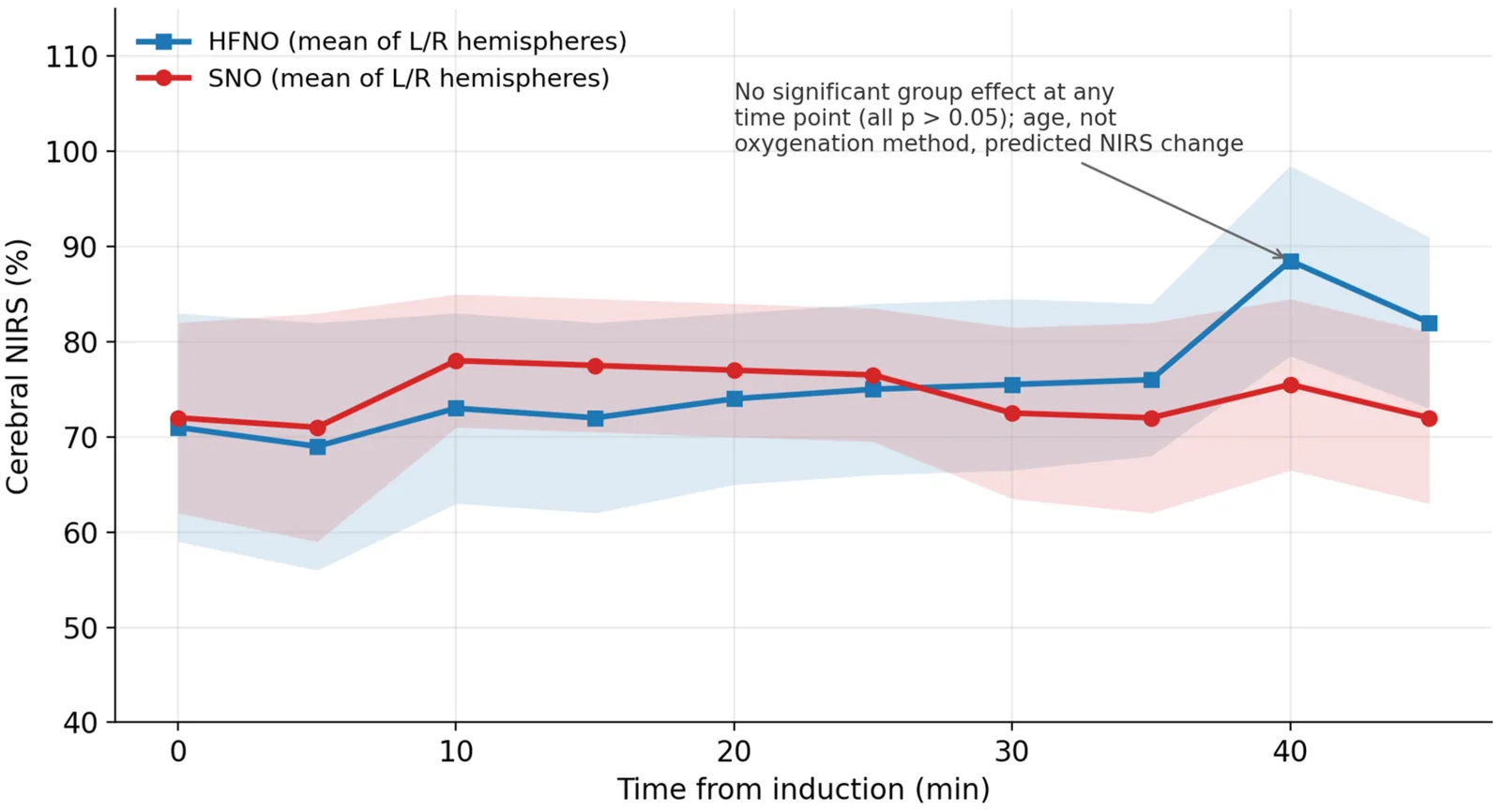
Changes in cerebral oxygen saturation (NIRS) over time by oxygenation group (left and right hemispheres averaged for clarity). Data are expressed as mean ± SD. NIRS: near-infrared spectroscopy; HFNO: high-flow nasal oxygen; SNO: standard nasal oxygen.

The p-spline model for BIS change did not reveal a significant non-linear relationship with SpO_2_ (*p* = 0.14), and the spline model provided no additional contribution over the linear model. Similarly, for both right- and left-sided NIRS changes, SpO_2_ was not a significant predictor; however, age was significantly associated with both BIS change (*p* = 0.022) and NIRS changes (right: *p* = 0.032; left: *p* = 0.004). Changes in NIRS over the procedure for both hemispheres and oxygenation groups are depicted in Figure 3.

### 3.6. Hemodynamic Parameters

No statistically significant differences were observed between the two groups in hemodynamic parameters throughout the procedure, although the HFNO group appeared clinically more stable based on graphical inspection.

## 4. Discussion

In this study, we compared the effects of HFNO and SNO therapy on hypoxia prevention in minor and moderate burn patients undergoing deep sedation. HFNO was associated with significantly more stable and effective maintenance of oxygenation throughout the procedure.

We elected to compare HFNO against standard nasal cannula oxygen rather than against other conventional methods such as a Venturi mask because nasal cannula oxygen is the default delivery method used in our burn unit for supine sedation procedures that do not require oral access, and because a full face mask can interfere with visual assessment of the airway, verbal contact with a lightly to moderately sedated patient, and rapid jaw-thrust maneuvers considerations of particular importance during burn dressing and debridement procedures.

Hypoxia is the most common adverse event during procedural sedation, with an incidence reported between 10% and 70% depending on its definition [8]. This wide range is attributed to differences in patient demographics, sedation methods, and varying hypoxia thresholds [6,9].

In a multicenter study of 7952 sedated patients, Mason et al. identified hypoxia as the most common adverse event (7.8 per 10,000 sedation encounters), with airway obstruction occurring at 5.42 per 10,000 [10].

Lin et al. compared HFNO and SNO in 1994 patients undergoing gastroscopy under propofol sedation. Hypoxia was observed in 8.4% of the SNO group, while no cases occurred in the HFNO group [11]. Nay et al. similarly found desaturation in 9.4% vs. 33.5% and airway maneuver requirements in 11.1% vs. 32.4% for HFNO and SNO groups, respectively, in a high-risk patient cohort [5]. In a meta-analysis of 2633 patients across six studies, Liu et al. confirmed that HFNO reduced desaturation risk and yielded higher minimum SpO_2_ compared with SNO, although no difference in airway interventions or complications was noted [12]. In contrast, Klotz and Shukla reported no benefit of HFNO over SNO in pediatric and adult gastrointestinal endoscopy populations, respectively [13,14].

More recent, larger-scale evidence corroborates these findings. A 2023 systematic review and meta-analysis of 17 randomized trials (2024 HFNO and 2037 control patients) by Thiruvenkatarajan et al. confirmed that HFNO reduces the incidence of hypoxaemia during procedural sedation across a range of clinical settings. A 2024 trial sequential analysis by Chen et al. subsequently concluded that the cumulative evidence for this benefit has reached a level of statistical robustness that makes it unlikely to be reversed by further conventionally sized trials. These larger and more recent analyses are consistent with, and lend additional weight to, the findings of the present study [15,16].

The use of HFNO has also been evaluated in bronchoscopy settings, consistently showing reduced hypoxia rates [17,18,19,20]. Shyamsunda et al. found significantly lower hypoxia rates with HFNO in 960 patients undergoing hysteroscopy under sedation [21].

Data on HFNO in burn patients are scarce. Akın et al. reported successful use of HFNO during procedural sedation in patients with major burn–related ARDS, maintaining SpO_2_ above 95% in both cases [7]. Coletta et al. used HFNO in 14 burn patients undergoing enzymatic debridement; desaturation occurred in only two patients and resolved with jaw-lift maneuver alone [4].

In our study, the mean SpO_2_ was 4.68 units higher in the HFNO group than in the SNO group. This difference remained statistically significant after adjustment for BMI, ASA score, procedure duration, smoking status, burn type, and percentage of burned surface area. Jaw thrust was required in 84% of SNO patients vs. only 2.6% in the HFNO group, and flow/FiO_2_ adjustments were similarly far more frequent in the SNO group. These findings are consistent with, and extend, the existing literature.

BIS monitoring was used to standardize sedation depth across groups. Although BIS values tended to be lower and NIRS values higher in the HFNO group, neither difference reached statistical significance, suggesting comparable anesthetic depth. Importantly, both BIS and NIRS percentage changes were independently associated with patient age rather than oxygenation method, in line with the findings of Velegraki et al. and Carlson et al. [22,23]. These results highlight the potential added value of NIRS in detecting subtle cerebral oxygenation changes not captured by conventional monitoring [23].

No significant hemodynamic differences were observed between groups, consistent with the findings of Mazzeffi et al. [24] and Carron et al. [25].

### Limitations

This study has several limitations. First, the oxygenation method was not randomly assigned but followed the pre-specified, non-randomized rule described in Section 2.1; although baseline demographic, comorbidity, and burn characteristics were comparable between groups, the possibility of unmeasured selection bias cannot be fully excluded. Second, several variables plausibly associated with sedation-related hypoxia, airway anatomy (e.g., Mallampati score), neck circumference, history of obstructive sleep apnea, and baseline pulmonary function were not systematically recorded or adjusted for in this study and should be incorporated into future work. Third, our definition of severe hypoxia (SpO_2_ < 75%) is stricter than the 80–85% thresholds used in much of the procedural sedation literature; although this reflects the SpO_2_ level at which bag-mask ventilation was clinically required in our protocol, it limits direct numerical comparison of severe-hypoxia incidence with prior studies. Fourth, the inability to measure end-tidal or transcutaneous CO_2_ precluded assessment of ventilatory, as opposed to oxygenation, status a particularly important gap because HFNO can preserve SpO_2_ despite rising CO_2_ and mask evolving hypoventilation; this represents an important safety consideration that future controlled studies of HFNO in sedated, spontaneously breathing patients should specifically address. Fifth, HFNO equipment, disposables, and heated-humidified circuits are considerably more costly than standard nasal cannulae and require staff familiarity with the device; we did not perform a formal cost-effectiveness analysis, and equipment cost, staffing requirements, and device availability should be weighed against the observed clinical benefit, particularly in resource-limited burn centers. Sixth, patients with facial burns, inhalation injury, major burns, or ASA ≥ IV disease were excluded from this study, which limits the generalizability of our findings to the more severely affected burn population that may be at greatest risk of sedation-related respiratory compromise. Seventh, exploratory subgroup analyses, for example, BMI ≥ 30 kg/m^2^, smoking status, ASA III, higher burn percentage, older age, or higher propofol dose, could not be performed within the scope of the present dataset and analysis but represent a natural next step that may help identify which patients benefit most from HFNO. Finally, and most fundamentally, the observational design of this study precludes definitive causal inference; our findings should therefore be interpreted as hypothesis-generating, and confirmation in adequately powered randomized controlled trials is warranted before these results can inform routine clinical practice.

## 5. Conclusions

HFNO significantly reduced the incidence of hypoxia and the need for airway interventions compared with SNO during deep sedation in minor and moderate burn patients. HFNO therefore appears to be a promising oxygenation strategy that may reduce sedation-related respiratory adverse events in this population. However, because these findings derive from a non-randomized observational study, they should be interpreted as hypothesis-generating rather than practice-defining, and confirmation in adequately powered randomized controlled trials is warranted before HFNO can be recommended for routine practice in burn sedation. In the meantime, these findings may usefully inform the design of such future trials.

## Figures and Tables

**Table 1:** Comparison of demographic characteristics.

Variable	HFNO (n = 38)	SNO (n = 38)	*p*-Value
Age (years)	46.3 ± 15.7	42.3 ± 13.0	0.5
Sex—Female/Male	14 (37%)/24 (63%)	15 (39%)/23 (61%)	0.8
Weight (kg)	75.8 ± 12.2	78.5 ± 13.1	0.3
Height (cm)	168.4 ± 9.5	168.3 ± 9.3	>0.9
BMI (kg/m^2^)	26.8 ± 4.2	27.8 ± 4.7	0.6
ASA I/II/III	5 (13%)/29 (76%)/4 (11%)	8 (21%)/29 (76%)/1 (2.6%)	0.4

Data are presented as mean ± SD or n (%). *p*-values from Wilcoxon rank-sum test, Pearson’s χ^2^, or Fisher’s exact test as appropriate. HFNO: high-flow nasal oxygen; SNO: standard nasal oxygen; BMI: body mass index; ASA: American Society of Anesthesiologists.

**Table 2:** Comparison of comorbidities and smoking status.

Comorbidity	HFNO (n = 38)	SNO (n = 38)	*p*-Value
HT	9 (24%)	5 (13%)	0.2
DM	4 (11%)	5 (13%)	>0.9
Hyperthyroidism	4 (11%)	2 (5.3%)	0.7
CAD	2 (5.3%)	0 (0%)	0.5
Asthma	2 (5.3%)	2 (5.3%)	>0.9
COPD	2 (5.3%)	0 (0%)	0.5
Rheumatologic disease	1 (2.6%)	3 (7.9%)	0.6
Neurological disease	3 (7.9%)	0 (0%)	>0.9
BPH	1 (2.6%)	0 (0%)	0.2
HBsAg positive	2 (5.3%)	0 (0%)	0.5
Smoking	22 (58%)	17 (45%)	0.3

Data are presented as n (%). HT: hypertension; DM: diabetes mellitus; CAD: coronary artery disease; COPD: chronic obstructive pulmonary disease; BPH: benign prostatic hyperplasia. *p*-values from Pearson’s χ^2^ or Fisher’s exact test.

**Table 3:** Comparison of burn type, burn percentage, and procedure type.

Variable	HFNO (n = 38)	SNO (n = 38)	*p*-Value
Flame burn	13 (34%)	13 (34%)	0.6
Scald burn	17 (45%)	17 (45%)	
Chemical burn	4 (11%)	3 (7.9%)	
Electrical burn	2 (5.3%)	0 (0%)	
Contact burn	2 (5.3%)	5 (13%)	
Burn percentage, median (Q1–Q3)	8.0 (3.0–12.0)	6.0 (4.0–10.0)	>0.9
Escharectomy	35 (92%)	35 (92%)	>0.9
Graft	3 (7.9%)	3 (7.9%)	

Data are presented as n (%) or median (Q1–Q3). *p*-values from Fisher’s exact test or Wilcoxon rank-sum test.

**Table 4:** Comparison of total drug dose and procedure duration.

Variable	HFNO (n = 38)	SNO (n = 38)	*p*-Value
Total propofol dose (mg)	205 ± 94	188 ± 91	0.7
Procedure duration (min), median (Q1–Q3)	28 (25–34)	26 (22–33)	0.11

Data are presented as mean ± SD or median (Q1–Q3). *p*-values from Wilcoxon rank-sum test.

**Table 5:** Comparison of SpO_2_ categories between groups.

SpO_2_ Category	HFNO (n = 38)	SNO (n = 38)	*p*-Value
SpO_2_ ≥ 90%	4 (11%)	30 (79%)	<0.001
75% < SpO_2_ < 90%	0 (0%)	26 (68%)	<0.001
SpO_2_ ≤ 75%	0 (0%)	10 (26%)	<0.001

Data are presented as n (%). *p*-values from Fisher’s exact test.

**Table 6:** Comparison of airway interventions between groups.

Intervention	HFNO (n = 38)	SNO (n = 38)	*p*-Value
Jaw thrust	1 (2.6%)	32 (84%)	<0.001
Increase in flow rate	4 (11%)	32 (84%)	<0.001
Increase in FiO_2_	2 (5.3%)	32 (84%)	<0.001
Interruption of procedure	0 (0%)	4 (11%)	0.12
Spasm during procedure	0 (0%)	2 (5.3%)	0.5

Data are presented as n (%). *p*-values from Fisher’s exact test. FiO_2_: fraction of inspired oxygen.

## Data Availability

The data presented in this study are available on request from the corresponding author. The data are not publicly available due to privacy restrictions.
